# Development of Glycoconjugated MAGL Inhibitors with Glucose-Dependent Antiproliferative Activity

**DOI:** 10.3390/ijms27062666

**Published:** 2026-03-14

**Authors:** Giulia Bononi, Federica Bertini, Samuele Masoni, Miriana Di Stefano, Rossella Mosca, Francesca Felice, Giovanni Signore, Filippo Minutolo, Carlotta Granchi, Tiziano Tuccinardi, Valeria Di Bussolo

**Affiliations:** 1 Department of Pharmacy, University of Pisa, Via Bonanno 6, 56126 Pisa, Italy; giulia.bononi@unipi.it (G.B.); f.bertini15@studenti.unipi.it (F.B.); samuele.masoni@phd.unipi.it (S.M.); miriana.distefano@farm.unipi.it (M.D.S.); filippo.minutolo@unipi.it (F.M.); tiziano.tuccinardi@unipi.it (T.T.); valeria.dibussolo@unipi.it (V.D.B.); 2 Center for Instrument Sharing University of Pisa (CISUP), Lungarno Pacinotti 43, 56126 Pisa, Italy; 3 Biochemistry Unit, Department of Biology, University of Pisa, Via S. Zeno 51, 56127 Pisa, Italy; r.mosca@studenti.unipi.it (R.M.); francesca.felice@unipi.it (F.F.); giovanni.signore@unipi.it (G.S.); 4 Institute of Clinical Physiology, National Research Council, 56124 Pisa, Italy

**Keywords:** monoacylglycerol lipase, MAGL, MAGL inhibitors, glucoconjugates, glucoconjugates MAGL inhibitors, cancer

## Abstract

Monoacylglycerol lipase (MAGL) is a key regulator of lipid signaling networks implicated in tumor progression and represents an attractive anticancer target. To combine MAGL inhibition with potentially enhanced uptake by highly glycolytic cancer cells, we designed glycoconjugated analogs of a *N*-benzoylpiperidine MAGL inhibitor scaffold bearing a glucopyranose unit. An alkyne-functionalized benzoylpiperidine intermediate was prepared and coupled to azido sugars through a CuAAC “click” reaction to afford two triazole-linked glycoconjugates. In a colorimetric assay on human MAGL, the new compounds **17** and **18** inhibited the enzyme with IC_50_ values of 43.3 and 68.8 μM, respectively, confirming compatibility with MAGL inhibition albeit with reduced potency versus reference triazole-substituted benzoylpiperidine **13** (IC_50_ = 4.1 μM). In PANC-1 pancreatic cancer cells, both glycoconjugates were inactive in high-glucose medium, but displayed antiproliferative activity under low-glucose conditions (GI_50_ **17** = 129 μM; GI_50_ **18** = 12 μM), consistent with glucose-dependent uptake/competition. Overall, these first-in-class MAGL-targeting glycoconjugates provide a starting point for optimizing dual MAGL inhibition and metabolically driven cellular selectivity.

## 1. Introduction

Cancer is a complex disease characterized not only by uncontrolled proliferation but also by profound metabolic reprogramming, which enables tumor cells to survive and thrive under diverse microenvironmental stresses [1,2,3,4]. Among the hallmarks of cancer metabolism, alterations in glucose and lipid pathways are particularly critical [5,6,7,8,9]. Cancer cells often exhibit the Warburg effect, a metabolic shift in which glucose is preferentially converted to lactate even in the presence of sufficient oxygen [10,11]. This phenomenon provides rapid ATP production and generates biosynthetic intermediates necessary for nucleotide, aminoacid, and lipid synthesis, supporting accelerated growth and proliferation. A key feature of this reprogramming is the upregulation of glucose transporters (GLUTs), especially GLUT1, which enhances glucose uptake and contributes to sustain tumor survival and growth [12,13]. Notably, GLUT1 overexpression has been reported in pancreatic ductal adenocarcinoma, including commonly used cell models such as PANC-1, where it is associated with increased glucose uptake and aggressive tumor behavior [14,15,16].

Consistent with this metabolic phenotype, increasing evidence suggests that the elevated glucose uptake of cancer cells can be exploited to enhance the intracellular delivery of therapeutic and diagnostic agents. In this context, the conjugation of bioactive molecules to glucose or glucose-mimicking moieties has emerged as an effective strategy to promote preferential accumulation in highly glycolytic cancer cells through the engagement of overexpressed glucose transporters [17,18,19].

In parallel, lipid metabolism is profoundly rewired in many cancers and has attracted increasing interest in recent years [7,8,9,20]. Fatty acids are essential for membrane biogenesis [21,22,23,24,25] and also act as signaling molecules that promote oncogenic pathways and metastatic progression [8,26].

Monoacylglycerol lipase (MAGL) plays a central role in lipid metabolism and endocannabinoid signaling, by catalyzing the hydrolysis of monoacylglycerols into free fatty acids and glycerol, including endocannabinoid 2-arachidonoylglycerol (2-AG). Elevated MAGL activity has been associated with increased tumor aggressiveness, enhanced invasiveness, and poor clinical outcomes in multiple cancer types [27,28]. This has positioned MAGL as a highly attractive therapeutic target, motivating extensive research into the development of selective inhibitors capable of modulating lipid-dependent oncogenic signaling [29,30].

**Figure 1:**
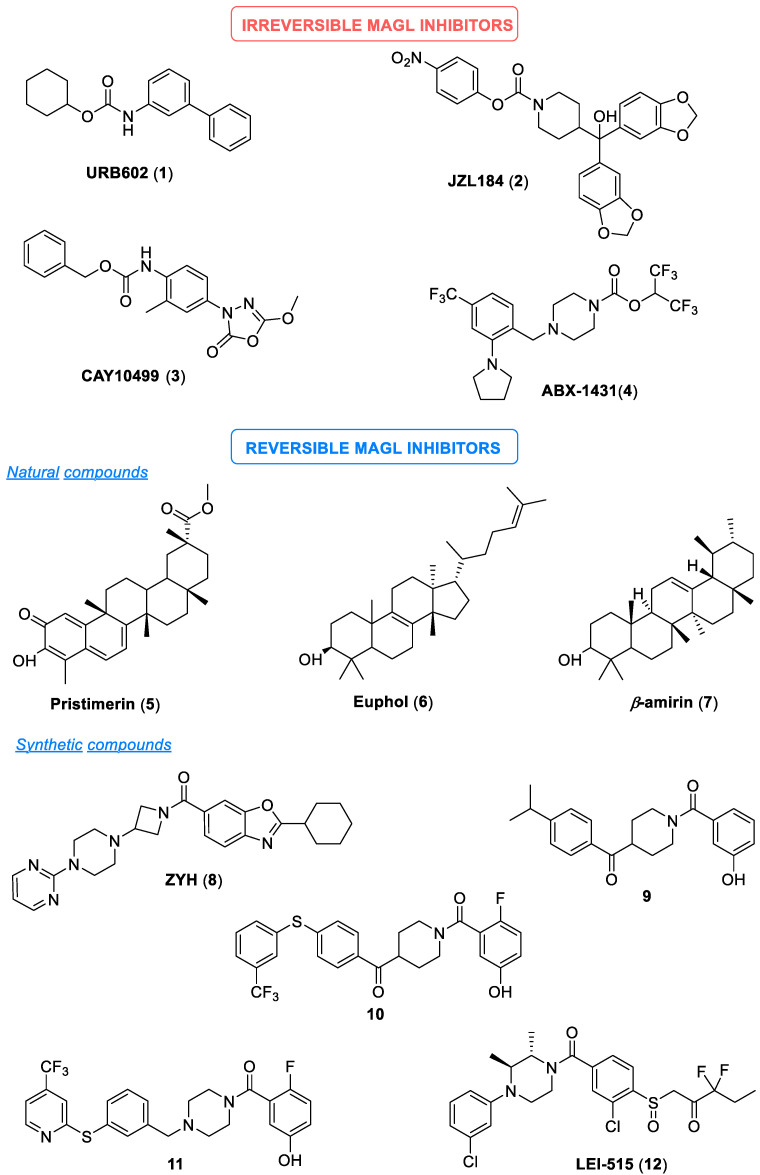
Structures of some of the most representative irreversible and reversible MAGL inhibitors.

Over the years, research on MAGL has led to the discovery of both irreversible and reversible inhibitors [30]. Irreversible inhibitors such as URB602, JZL184, CAY10499 and ABX-1431 (compounds **1**–**4**, Figure 1) covalently modify the enzyme and generally display high potency and selectivity [31,32,33,34]. However, they carry the risk of adverse effects, including receptor desensitization, physical dependence and tolerance in the endocannabinoid system [35,36]. Reversible inhibitors, on the other hand, allow transient inhibition of MAGL activity, maintaining physiological enzyme levels while reducing the likelihood of undesired side effects. Several classes of reversible inhibitors have been reported to date, ranging from early natural triterpenoids with limited selectivity, such as pristimerin, euphol and *β*-amyrin (compounds **5**–**7**, Figure 1) [37,38], to advanced synthetic scaffolds such as ZYH (compound **8**), **9**, **10**, **11** and LEI-515 (compound **12**, Figure 1), which show notable potency and good selectivity over other endocannabinoid system enzymes [39,40,41,42,43].

Our group has significantly contributed to this field, developing various classes of potent reversible MAGL inhibitors, particularly those based on the benzoylpiperidine scaffold [40,41,44,45,46]. The benzoylpiperidine core combines favorable metabolic stability with chemical versatility, supporting the design of effective MAGL inhibitors. These compounds have demonstrated remarkable potency and selectivity toward MAGL, providing valuable chemical tools to modulate MAGL function and explore its therapeutic potential.

Building on this expertise, we hypothesized that linking a glucose-mimicking moiety to a benzoylpiperidine scaffold could lead to the identification of a novel class of MAGL inhibitors displaying an enhanced uptake in glycolytic cancer cells, while preserving enzymatic inhibition. In this study, we report the design, synthesis, and biological evaluation of a novel series of glycoconjugated MAGL inhibitors, in which a benzoylpiperidine core is functionalized with glucopyranose portions. To the best of our knowledge, these compounds represent the first glycoconjugated inhibitors specifically targeting MAGL, offering a strategy to combine enzyme inhibition with selective cellular uptake in cancer cells.

### Rationale Design

In recent years, our research group has developed several reversible MAGL inhibitors based on benzoylpiperidine scaffolds, which display notable potency and selectivity toward MAGL [40,41,44,45,46]. The benzoylpiperidine core is well-known as a privileged structure in the development of new drugs, owing to its favorable metabolic stability which makes it a versatile and reliable chemical frame to be exploited in drug design [47].

**Figure 2:**
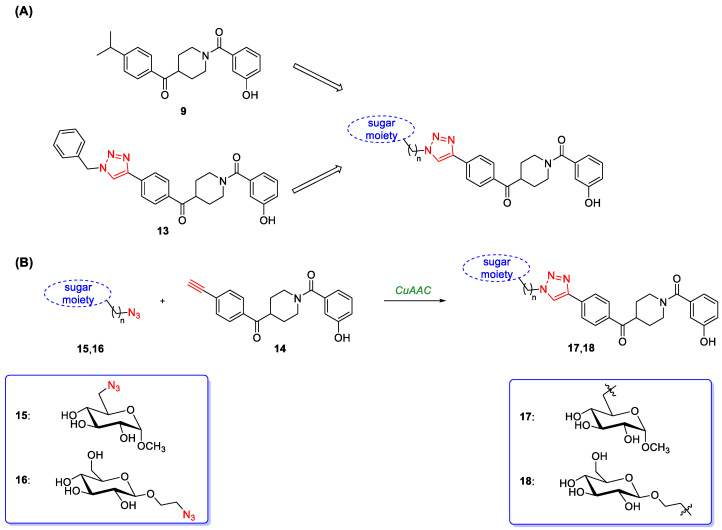
(**A**) Structure of reference compounds **9** and **13** [40,46] and general structure of the newly designed glycoconjugated MAGL inhibitors, in which the carbohydrate portion is reported in blue and the triazole ring is shown as the linker in red. (**B**) Copper-catalyzed azido-alkyne cycloaddition (CuAAC) between the appropriate azido sugars **15**, **16** (the azido group is highlighted in red) and the benzoylpiperidine derivative bearing a terminal alkyne group (compound **14**, the alkyne moiety is in red) to obtain the desired glycoconjugated MAGL inhibitors **17**, **18**.

In the present work, we aimed to develop a new series of MAGL inhibitors characterized by a benzoylpiperidine core which is functionalized with a sugar moiety, specifically a glucopyranose unit, with the goal of enhancing cellular uptake in cancer cells (the general structure of glucoconjugates is reported in Figure 2A). The glucopyranose unit was selected as a glucose mimic, since it has been widely demonstrated that it can be potentially recognized by the glucose transporters (GLUTs) [48]. Starting from the known MAGL inhibitors **9** and **13** [40,46], the presence of a triazole moiety in **13** prompted its use as a versatile handle for further functionalization. This triazole functionality was used as a linker to introduce sugar moieties, leading to a new series of compounds represented by the general structure shown on the right (Figure 2A).

The synthetic strategy adopted to obtain the newly designed MAGL inhibitors first required the preparation of the benzoylpiperidine fragment bearing a terminal alkyne group (compound **14**, Figure 2B). The terminal triple bond was essential for linking the benzoylpiperidine scaffold to the azido-functionalized glucopyranose unit (compounds **15**, **16**, Figure 2B) through a copper-catalyzed azide–alkyne cycloaddition (CuAAC, Figure 2B). This approach successfully enabled the synthesis of the desired glycoconjugated MAGL inhibitors **17**, **18** (Figure 2B).

The detailed synthetic procedure, including each reaction step, will be described in the following section.

## 2. Results and Discussion

### 2.1. Chemistry

**Scheme 1:**
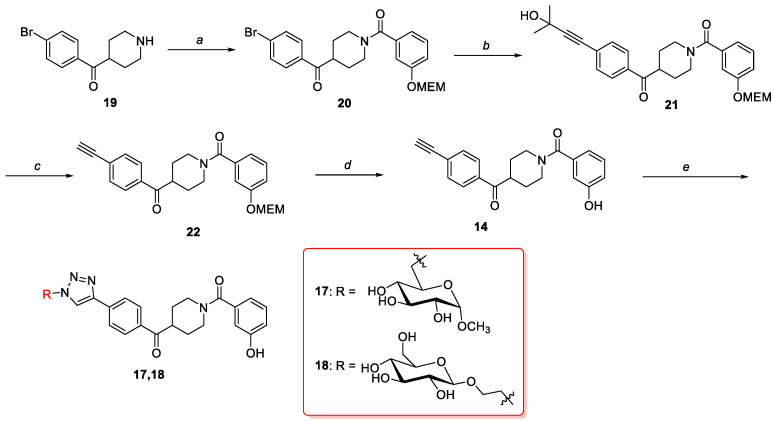
Synthesis of glycoconjugated MAGL inhibitors **17**,**18**. Reagents and conditions: (a) 3-((2-methoxyethoxy)methoxy)benzoic acid, HATU, DIPEA, anhydrous DMF, RT, 3 h [73%]; (b) MEBYNOL, Pd(OAc)_2_, PPh_3_, CuI, Et_3_N, anhydrous DMF, 90 °C, 4 h [71%]; (c) K_3_PO_4_, KOH, anhydrous toluene, 115 °C, 1 h [70%]; (d) 1 N aq. HCl, CH_3_OH, reflux, 2 h [87%]; (e) appropriate azido sugars **15** or **16**, CuSO_4_^.^5H_2_O, sodium ascorbate, H_2_O/*t*BuOH 1:1 *v*/*v*, 80–100 °C, 3–16 h [11–28%]. Bromo-derivative **19** and 3-((2-methoxyethoxy)methoxy)benzoic acid were synthesized according to the literature [41]. Azido sugars **15**, **16** were synthesized according to the literature [49,50,51,52].

These desired glycoconjugated MAGL inhibitors **17**, **18** were prepared through the synthetic pathway depicted in Scheme 1, starting from the key piperidine-based intermediate amine **19**, whose synthesis was previously reported in the literature [41]. In the first step, amine **19** [41] was subjected to an amide-coupling reaction with 3-((2-methoxyethoxy)methoxy)benzoic acid, also previously described by Bononi et al. [41]. The amide condensation was carried out in the presence of 1-[bis(dimethylamino)methylene]-1*H*-1,2,3-triazolo[4,5-*b*]pyridinium-3-oxide hexafluorophosphate (HATU) as the condensing agent, *N*,*N*-diisopropylethylamine (DIPEA) as the base and dry *N*,*N*-dimethylformamide (DMF) as the solvent affording intermediate **20** in excellent yields (step a, Scheme 1). Amide **20** was then subjected to a copper-catalyzed Sonogashira cross-coupling reaction with the terminal alkyne 2-methyl-3-butyn-2-ol (MEBYNOL), in the presence of palladium acetate, triphenylphosphine, with triethylamine as the base and DMF as the solvent (step b, Scheme 1). After 4 h of stirring, the desired propargylic alcohol derivative **21** was obtained in good yields (71%). Subsequently the 2-hydroxypropyl-protecting group of **21** was efficiently removed under basic conditions, reacting it with potassium phosphate and potassium hydroxide in refluxing dry toluene for 1 h, thus obtaining intermediate **22** in satisfactory yields (step c, Scheme 1). At this stage, the protecting group of *O*-MEM-substituted amide **22** was removed by a simple aqueous acidic hydrolysis by mild heating to afford the key benzoylpiperidine derivative **14**, bearing a terminal alkyne functionality, in excellent yields (step d, Scheme 1). With intermediate **7** readily available, attention was next directed toward the glucopyranosidic components required for the subsequent CuAAC reaction. The azido sugars **15** and **16** employed in the final CuAAC step (Figure 2) were synthesized following established and well-validated procedures previously reported in the literature [49,50,51,52]. Intermediate **14** was therefore subjected to a CuAAC with the appropriate azido sugars **15**, **16** leading to the formation of the final desired triazole-substituted glycoconjugated products **17** and **18** in low-to-moderate yields (step e, Scheme 1).

### 2.2. Biochemical Assays

The inhibitory activity of the newly synthesized glycoconjugated derivatives **17**, **18** toward human MAGL was evaluated using a colorimetric enzymatic assay based on the hydrolysis of 4-nitrophenyl acetate (4-NPA). In this assay, the MAGL-mediated cleavage of 4-NPA generates 4-nitrophenol, which can be quantified spectrophotometrically by monitoring the absorbance increase at 405 nm, enabling the calculation of the corresponding IC_50_ values. The inhibitory potencies of compounds **17**, **18** were compared with those of the previously reported benzoylpiperidine-based MAGL inhibitor **13** [46], used as reference compound (IC_50_ = 4.1 μM, Table 1).

Overall, the newly developed glycoconjugated MAGL inhibitors **17**, **18** exhibited lower inhibitory activity compared to the reference compounds, with IC_50_ values ranging from 43.3 to 68.8 μM. This reduction in inhibitory potency can be attributed to the increased polarity introduced by the sugar moiety, which is poorly tolerated by the enzyme’s active site. Indeed, MAGL features a long hydrophobic tunnel that normally accommodates the lipophilic acyl chain of the endogenous substrate 2-AG and is therefore less compatible with polar substituents. Regarding the position of conjugation on the glucopyranose moiety, no significant differences were detected between the derivative functionalized at C1 (compound **17**) and that functionalized at C6 (compound **18**). This indicates that, within this scaffold, the attachment site on the sugar ring does not markedly affect MAGL inhibitory activity.

To further investigate the potential activity of compounds **17** and **18** toward other hydrolases, their inhibitory activity was evaluated against the related enzyme fatty acid amide hydrolase (FAAH). Notably, both compounds showed no significant inhibition of this enzyme at the tested concentrations, with IC_50_ values exceeding 100 μM.

In summary, biochemical evaluation confirmed that incorporation of a glucopyranosyl unit onto the benzoylpiperidine scaffold is compatible with MAGL inhibition, although associated with reduced potency relative to the parent inhibitor **13** [46]. Importantly, these compounds represent the first examples of MAGL-targeting glycoconjugated inhibitors, providing a valuable foundation for the future design and optimization of more potent glycoconjugated MAGL inhibitors.

### 2.3. Modeling Studies

**Figure 3:**
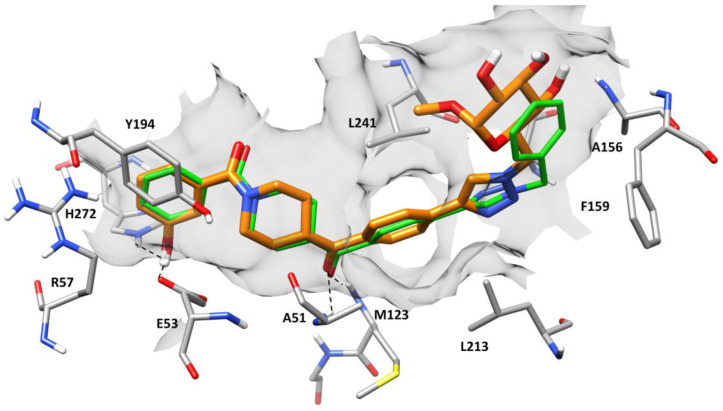
Docking pose of compound **17** (orange) superimposed to reference compound **13** (green) within the MAGL active site. Hydrogen bonds are depicted as dashed lines.

To further rationalize the MAGL inhibition potencies of the glycoconjugated inhibitors, docking calculations were carried out. The results indicate that the benzoylpiperidine core of the glycoconjugated derivatives adopts a binding mode closely superimposable to that of reference compound **13** within the MAGL active site, preserving the hydrogen-bond interactions with A51 and M123 in the oxyanion hole, as well as the characteristic hydrogen-bond network involving E53 and H272 (Figure 3).

At the same time, the glucopyranosyl of compound **17** moiety extends toward a more solvent-exposed region at the entrance of the catalytic pocket and does not establish significant compensatory hydrogen-bond interactions with the surrounding residues. Given the predominantly lipophilic character of this region, the introduction of a bulky and highly polar sugar moiety may be energetically disfavored. In particular, the carbohydrate fragment is expected to be strongly solvated in aqueous solution, and its partial transfer into a relatively hydrophobic environment may involve an unfavorable desolvation penalty that is not compensated by additional stabilizing protein–ligand interactions, ultimately resulting in reduced binding affinity and inhibitory potency.

### 2.4. Antiproliferative Activity

The efficacy of the synthesized compounds was assessed in vitro using PANC-1 cell line, a reportedly lipogenic tumor line [53] where inhibition of MAGL might have important effects on cell metabolism [54]. Furthermore, the effect of the inhibitors was tested both in high and low-glucose conditions, to verify potential changes due to the different metabolic statuses of the cell. As expected, the reference compound **13** was active at low-micromolar concentration both in high and low-glucose conditions (Table 2). Conversely, the two glycoside derivatives **17** and **18** show a measurable antiproliferative activity only in low-glucose conditions, with no measurable effect present in high-glucose conditions. This behavior might be rationalized by competition with glucose, which would hamper binding and internalization of the conjugated drug. In low-glucose conditions, the efficacy of the glycosylated compound **18** is comparable (three-fold increase in GI_50_, defined as the concentration required to inhibit cell growth by 50%) with that of the reference compound **13**, while compound **17** has markedly lower activity (GI_50_ greater than 100 micromolar). Although compounds **17** and **18** showed similar inhibitory potencies against purified *h*MAGL (Table 1; IC_50_ = 43.3 and 68.8 µM, respectively), they exhibited markedly different antiproliferative activities in the cell-based assay (Table 2; compound **17**: GI_50_ = 129 µM; compound **18**: GI_50_ = 12 µM). This suggests that the glycoside unit plays an important role in promoting differential internalization through interactions with membrane transporters in glycolytic cancer cells, and that this effect may strongly depend on glycoside structure. In particular, functionalization of the glucopyranose moiety at the anomeric C1 position with *β*-stereochemistry proved to be better tolerated in terms of antiproliferative effect, which can possibly be ascribed to higher cellular uptake. In line with the literature data on glucose conjugation and GLUT-mediated transport, differences in the position and nature of substitution on the glucose scaffold are known to be tolerated by glucose transporters, although bulky substituents may lead to a reduction in transporter affinity rather than complete loss of transport capability. Within this framework, the different glycosylation patterns of compounds **17** and **18** may result in different degrees of transporter compatibility and cellular uptake efficiency, thereby contributing to the distinct antiproliferative activities observed under low-glucose conditions. We are aware that our data currently cannot demonstrate with certainty the involvement of a glucose transporter in this preferential accumulation in cancer cells. Nevertheless, the fact that the antiproliferative effect of gluco-conjugated analogs **17** and, in particular, **18** emerges only under low-glucose conditions (where competition with extracellular glucose is less intense) could constitute an indirect indication that the cellular uptake of these compounds may, at least in part, follow shared uptake pathways with those of glucose itself.

**Figure 4:**
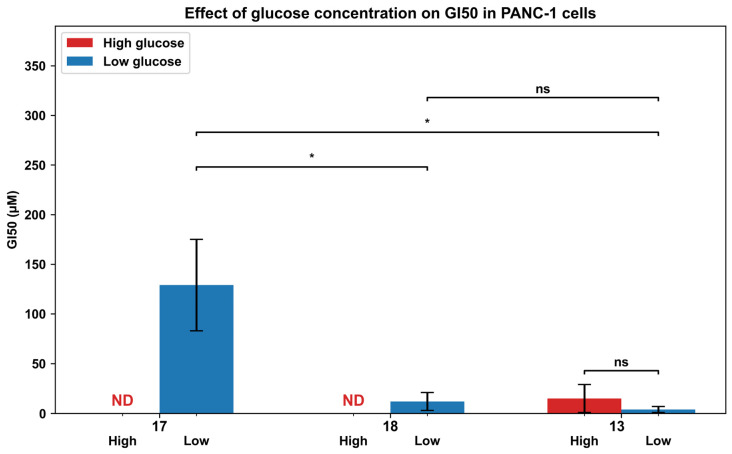
Comparison of antiproliferative activity (GI_50_) of PANC-1 cell line in different glucose conditions. GI_50_ values of compounds **17**, **18** and **13** in PANC-1 cells cultured under high- and low-glucose conditions. Data are expressed as mean ± SD of three independent experiments. ND indicates that the GI_50_ value was higher than the maximum tested concentration (>1000 µM). Statistical comparisons between high- and low-glucose conditions for each compound, as well as between compounds within the same glucose condition, were performed using Welch’s *t*-test. Significance levels are indicated as *p* < 0.05 (*) and not significant (ns).

Both glycosides are characterized by a lower effectiveness in terms of growth inhibition compared with the reference MAGL inhibitor **13**. These assays are only preliminary tests, which were essential to confirm a basal level of antiproliferative activity for these novel glycoconjugated MAGL inhibitors. However, the intriguing internalization features of **18** might warrant additional investigation due to the preferential internalization in low-glucose conditions, which are reportedly typical of solid tumor microenvironment [55,56,57]. Comparison between the efficacy of the compounds in high- and low-glucose conditions (Figure 4) highlights the relative insensity of compound **13** to glucose conditions (difference between GI_50_ not significant) and the significant difference between the two glycosylated compounds **17** and **18**. In particular, measured GI_50_ for compound **18** in low-glucose conditions was not significantly different from that of reference drug **13**. To further support this preliminary mechanistic investigation, we tested the same compounds in the MiA PaCa-2 cell line, which shows markedly different metabolic profiles, being one of the most glycolytic commercial lines [53]. The underlying idea was that the highly glycolytic metabolism should make them less susceptible to the treatment with reference and glycosylated derivatives. As expected, the efficacy of all the tested compounds was markedly lower, with the two glycosylated derivatives being ineffective at all tested concentrations and the reference compound **13** showing only a modest cytotoxicity at high concentrations (Table 2). Finally, we tested the selectivity of the synthesized compounds with respect to a physiologic control cell line. To this end, we assessed the cytotoxicity towards a physiologic pulmonary fibroblast cell line. We observed an eight-fold increase in IC_50_ of the untargeted reference compound **13**, while the effect for glycosylated derivatives was not measurable at all tested concentrations (Table 2). This suggests that the glycoside motif might cause a significant boost in selectivity of the drug. The possibility to modulate internalization and, hence, cytotoxicity, depending on glucose level, might open the way to the use of more selective MAGL inhibitors.

## 3. Materials and Methods

### 3.1. Synthesis: General Procedures and Materials

All solvents and reagents were used as obtained commercially, without additional purification. Chromatographic separations were carried out on silica gel columns by flash chromatography (Kieselgel 40, 0.040–0.063 mm; Merck, Darmstadt, Germany). Reaction progress was monitored by thin layer chromatography (TLC) on Merck aluminum silica gel (60 F254) sheets, visualized under a UV lamp. Solvent removal was performed in vacuo with a rotary evaporator. Anhydrous sodium sulfate was always employed as the drying agent.

Proton (^1^H) and carbon (^13^C) NMR spectra were obtained with a Bruker Avance III 400 MHz spectrometer (Bruker, Billerica, MA, USA) using the specified deuterated solvents. Chemical shifts are given in parts per million (ppm) (δ relative to residual solvent peak for ^1^H and ^13^C). ^1^H-NMR spectra are reported in the following order: multiplicity and number of protons. Standard abbreviation indicating multiplicity was used as follows: s = singlet; d = doublet; dd = doublet of doublets; dq = double of quartets; t = triplet; m = multiplet; bm = broad multiplet.

HPLC analyses confirmed that all final compounds tested in biological tests had purity ≥ 95%. HPLC analyses were performed using a Shimadzu HPLC system (Shimadzu Corporation, Kyoto, Japan). Reverse-phase analytical HPLC was conducted using a Kinetex EVO C18 column (5 µm, 150 mm × 4.6 mm, Phenomenex, Inc., Torrance, California, USA) with UV analysis (λ = 254 nm). Compounds **17** and **18** were analyzed using the following method: eluent A, water; eluent B, CH_3_CN; after 6 min at 15% B, a gradient from 15% to 25% B is formed in 2 min and held at 25% B for 12 min; the flow rate is 1 mL/min.

The ESI-MS spectra were recorded by direct injection at a 5 μL min^−1^ flow rate in an Orbitrap high-resolution mass spectrometer (Thermo, San Jose, CA, USA), equipped with an HESI source. The working conditions were as follows: negative polarity; spray voltage of 3.0 kV; capillary temperature at 320 °C; S-lens RF level 50; the sheath gas was set at 10; the auxiliary gas was set at 3 (arbitrary units). For acquisition and analysis, Xcalibur 4.2 software (Thermo) was used. For spectra acquisition, a nominal resolution (at *m*/*z* 200) of 140,000 was used.

Reported yields correspond to isolated and purified products derived from non-optimized procedures. Bromo-derivative **19** and 3-((2-methoxyethoxy)methoxy)benzoic acid were synthesized according to the literature [41]. Azido sugars **15**, **16** were synthesized according to the literature [49,50,51,52].

#### 3.1.1. Procedure for the Synthesis of Compound **20**

HATU (1.05 equiv) was added to a solution of 3-((2-methoxyethoxy)methoxy)benzoic acid [41] (1 equiv) in dry DMF (12.7 mL), then DIPEA (four equiv) was added dropwise. The resulting mixture was stirred at room temperature for 30 min and then amine **19** [41] (740 mg, 1 equiv) was added and left under stirring at room temperature for 3 h. After the stated duration, the residue was diluted with water and extracted with EtOAc. The organic layer was repeatedly washed with brine, dried over anhydrous sodium sulfate and the solvent was removed under reduced pressure. The residue was purified with a flash column chromatography (silica gel, mixture of *n*-hexane/ethyl acetate from 4:6 to 3:7) and pure fractions containing the desired compound were evaporated to dryness affording amide **20**.

(4-(4-Bromobenzoyl)piperidin-1-*yl*)(3-((2-methoxyethoxy)methoxy)phenyl)methanone (**20**): dark yellow oil. TLC: *n*-hexane/ethyl acetate 4:6. 73% yield from **19** [41]. ^1^H-NMR (CDCl_3_) δ (ppm): 1.72–1.86 (bm, 3H), 1.90–2.11 (bm, 1H), 2.95–3.25 (bm, 2H), 3.37 (s, 3H), 3.42–3.55 (m, 1H), 3.52–3.59 (m, 2H), 3.77–3.95 (bm, 1H), 3.79–3.86 (m, 2H), 4.59–4.76 (bm, 1H), 5.28 (s, 2H), 7.03 (d, 1H, *J* = 7.5 Hz), 7.06–7.14 (m, 2H), 7.32 (t, 1H, *J* = 7.8 Hz), 7.63 (d, 2H, *J* = 8.3 Hz), 7.81 (d, 2H, *J* = 8.5 Hz).

#### 3.1.2. Procedure for the Synthesis of Compound **21**

A solution of Pd(OAc)_2_ (0.05 equiv) and triphenylphosphine (0.25 equiv) in dry DMF (0.4 mL) was stirred at RT under argon for 10 min. After that period, aryl-bromide **20** (235 mg,1 equiv), copper iodide (0.05 equiv), 2-methyl-3-butyn-2-ol (1.2 equiv) and Et_3_N (1.6 mL) were sequentially added. The mixture was stirred at 90 °C for 4 h. After being cooled to RT, the mixture was diluted with water and extracted several times with EtOAc. The combined organic phase was washed with brine, dried over anhydrous sodium sulfate and concentrated. The crude product was purified by flash chromatography (silica gel, mixture of CH_3_Cl/CH_3_OH 98:2) to yield the desired product **21**.

(4-(4-(3-Hydroxy-3-methylbut-1-yn-1-*yl*)benzoyl)piperidin-1-*yl*)(3-((2-methoxyethoxy)methoxy)phenyl)methanone (**21**): yellow oil. TLC: CH_3_Cl/CH_3_OH 98:2. 71% yield from **20**. ^1^H-NMR (CDCl_3_) δ (ppm): 1.63 (s, 6H), 1.72–1.86 (bm, 3H), 1.93–2.07 (bm, 1H), 2.97–3.23 (bm, 2H), 3.37 (s, 3H), 3.44–3.49 (m, 1H), 3.50–3.60 (m, 2H), 3.77–3.93 (bm, 1H), 3.80–3.86 (m, 2H), 4.61–4.75 (bm, 1H), 5.28 (s, 2H), 7.03 (d, 1H, *J* = 7.5 Hz), 7.06–7.13 (m, 2H), 7.32 (t, 1H, *J* = 7.7 Hz), 7.51 (d, 2H, *J* = 8.2 Hz), 7.88 (d, 2H, *J* = 8.3 Hz).

#### 3.1.3. Procedure for the Synthesis of Compound **22**

Compound **21** (228 mg, 1 equiv) was suspended in anhydrous toluene (19.0 mL), then potassium hydroxide (1 equiv) and potassium phosphate (1 equiv) were added and the mixture was refluxed at 115 °C for 1 h. The reaction mixture was cooled to RT, filtered through a small pad of Celite and washed several times with ethyl acetate. Evaporation under vacuum of the filtrate afforded the crude product, which was subsequently purified by flash chromatography (silica gel, mixture of CH_3_Cl/CH_3_OH 98:2) to yield pure product **22**.

(4-(4-Ethynylbenzoyl)piperidin-1-*yl*)(3-((2-methoxyethoxy)methoxy)phenyl)methanone (**22**): red oil. TLC: CH_3_Cl/CH_3_OH 98:2. 70% yield from **21**. ^1^H-NMR (CDCl_3_) δ (ppm): 1.72–1.87 (bm, 3H), 1.93–2.08 (bm, 1H), 2.97–3.24 (bm, 2H), 3.27 (s, 1H), 3.37 (s, 3H), 3.45–3.59 (m, 1H), 3.49–3.61 (m, 2H), 3.75–3.94 (bm, 1H), 3.77–3.86 (m, 2H), 4.59–4.77 (bm, 1H), 5.28 (s, 2H), 7.03 (d, 1H, *J* = 7.4 Hz), 7.06–7.13 (m, 2H), 7.32 (t, 1H, *J* = 7.8 Hz), 7.59 (d, 2H, *J* = 8.3 Hz), 7.89 (d, 2H, *J* = 8.3 Hz).

#### 3.1.4. Procedure for the Synthesis of Compound **14**

A total of 1 N aqueous solution of HCl (6.6 mL) was added to a solution of compound **22** (280 mg, 1 equiv) in methanol (6.6 mL). The resulting mixture was refluxed until the starting material was consumed (TLC). Then the mixture was quenched with H_2_O, and the organic solvent was removed under reduced pressure. The aqueous phase was extracted with EtOAc, washed with brine and dried over anhydrous sodium sulphate. The solvent was removed under reduced pressure. Purification was conducted by flash chromatography using a mixture of CHCl_3_/CH_3_OH 98:2 as eluent, affording pure compound **14**.

(4-(4-Ethynylbenzoyl)piperidin-1-*yl*)(3-hydroxyphenyl)methanone (**14**): white solid. TLC: CH_3_Cl/CH_3_OH 98:2. 87% yield from **22**. ^1^H-NMR (CD_3_OD) δ (ppm): 1.58–1.77 (bm, 2H), 1.78–1.90 (bm, 1H), 1.92–2.06 (bm, 1H), 3.01–3.18 (bm, 1H), 3.21–3.33 (bm, 1H), 3.69–3.78 (m, 1H), 3.77 (s, 1H), 3.78–3.88 (bm, 1H), 4.54–4.69 (bm, 1H), 6.77–6.91 (m, 3H), 7.27 (t, 1H, *J* = 7.9 Hz), 7.60 (d, 2H, *J* = 8.3 Hz), 8.00 (d, 2H, *J* = 8.2 Hz).

#### 3.1.5. General Procedure for the Synthesis of Final Compounds **17**, **18**

Alkyne **14** (1 equiv) and appropriate azido sugars **15**, **16** (1 equiv) were suspended in a 1:1 mixture of water and *tert*-butyl alcohol (0.46 mL for 0.114 mmol of starting material). A freshly prepared sodium ascorbate aqueous solution (0.1 equiv, 0.11 mL of water) was added, followed by copper (II) sulfate pentahydrate aqueous solution (0.01 equiv, 4 µL of water). The heterogeneous mixture was stirred vigorously at 80 or 100 °C in a sealed vial until consumption of the starting materials. The reaction mixture was cooled, diluted with water, and extracted with EtOAc. The combined organic extracts were washed with brine, dried with anhydrous sodium sulphate, filtered and concentrated. The crude product was purified by flash column chromatography (eluent mixtures of CH_2_Cl_2_ or CHCl_3_/CH_3_OH) to obtain the final triazole-substituted glycoconjugates.

(1-(3-Hydroxybenzoyl)piperidin-4-*yl*)(4-(1-(((*2R*,*3S*,*4S*,*5R*,*6R*)-3,4,5-trihydroxy-6-methoxytetrahydro-*2H*-pyran-2-*yl*)methyl)-*1H*-1,2,3-triazol-4-*yl*)phenyl)methanone (**17**): white solid. TLC: CH_2_Cl_2_/CH_3_OH 9:1. Eluent CH_2_Cl_2_/CH_3_OH 9:1. 28% yield from **14** and **15**. ^1^H-NMR (CD_3_OD) δ (ppm): 1.61–1.79 (bm, 2H, CH_2_ piperidine), 1.80–1.92 (bm, 1H, CH piperidine), 1.95–2.09 (bm, 1H, CH piperidine), 3.13–3.15 (bm, 1H, CH piperidine), 3.15 (s, 3H, OCH_3_), 3.15–3.18 (m, 1H, Glc-H), 3.35–3.40 (m, 1H, Glc-H), 3.63 (t, 1H, *J* = 9.2 Hz, Glc-H), 3.74–3.88 (bm, 2H, CH_2_ piperidine), 3.88–3.96 (m, 1H, Glc-H), 4.69 (dd, 2H, *J* = 14.2, 8.4 Hz, CH_2_-triazole), 4.66 (d, 1H, *J* = 3.7 Hz, anomeric Glc-H), 4.87–4.95 (m, 2H, CH_2_ piperidine), 6.79–6.83 (m, 1H, phenolic ring), 6.83–6.91 (m, 2H, phenolic ring), 7.28 (t, 1H, *J* = 7.9 Hz, phenolic ring), 8.00 (d, 2H, *J* = 8.4 Hz, *para*-disubstituted phenyl ring), 8.10 (d, 2H, *J* = 8.5 Hz, *para*-disubstituted phenyl ring), 8.50 (s, 1H, H-triazole). ^13^C-NMR (CD_3_OD) δ (ppm): 29.57 (CH_2_ piperidine), 30.17 (CH_2_ piperidine), 42.76 (CH_2_ piperidine), 44.20 (CH piperidine), 45.54 (CH_2_ piperidine), 52.66 (Glc-CH_2_), 55.51 (OCH_3_), 71.70 (Glc), 73.01 (Glc), 73.37 (Glc), 74.96 (Glc), 101.33 (anomeric Glc-C1), 114.45 (2C, *para*-disubstituted phenyl ring), 117.94 (CH phenolic ring), 118.51 (CH phenolic ring), 124.81 (CH phenolic ring), 126.78 (*para*-disubstituted phenyl ring), 130.37 (CH phenolic ring), 130.98 (CH-triazole), 136.46 (C-phenolic ring), 136.52 (*para*-disubstituted phenyl ring), 138.30 (2C, *para*-disubstituted phenyl ring), 147.42 (C-triazole), 158.99 (C-OH phenolic ring), 172.53 (amidic C=O), 203.17 (ketonic C=O). HPLC analysis (batch FB36 in the Supporting Information): retention time = 12.375; peak area 95% (254 nm). HRMS: *m*/*z* for C_28_H_31_N_4_O_8_ [M − H]^−^ calculated: 551.21419, found: 551.21484; for C_28_H_32_N_4_O_8_Cl [M + Cl]^−^ calculated: 587.19087, found: 587.19159.

(1-(3-Hydroxybenzoyl)piperidin-4-*yl*)(4-(1-(2-(((*2R,3R,4S,5S,6R*)-3,4,5-trihydroxy-6-(hydroxymethyl)tetrahydro-*2H*-pyran-2-*yl*)oxy)ethyl)-*1H*-1,2,3-triazol-4-*yl*)phenyl)methanone (**18**): white solid. TLC: CH_2_Cl_2_/CH_3_OH 9:1. Eluent CH_2_Cl_2_/CH_3_OH from 9:1 to 85:15. 11% yield from **14** and **16**. ^1^H-NMR (CD_3_OD) δ (ppm): 1.60–1.79 (bm, 2H, CH_2_ piperidine), 1.80–1.92 (bm, 1H, CH piperidine), 1.95–2.08 (bm, 1H, CH piperidine), 3.05–3.15 (bm, 1H, CH piperidine), 3.16–3.24 (m, 1H, one of the two diasterotopic Alk-CH_2_-O-Glc), 3.25–3.31 (m, 3H, one of the two diasterotopic Alk-CH_2_-O-Glc and 2 × Glc-H), 3.61–3.69 (m, 1H, Glc-H), 3.73–3.85 (m, 2H, CH_2_ piperidine), 3.86–3.90 (m, 1H, Glc-H), 4.02–4.11 (m, 1H, Glc-H), 4.27–4.33 (m, 1H, Glc-H), 4.35 (d, 1H, *J* = 7.8 Hz, anomeric Glc-H1), 4.57–4.68 (bm, 1H, CH piperidine), 4.69–4.75 (m, 2H, CH_2_-triazole), 6.80–6.83 (m, 1H, phenolic ring), 6.84–6.90 (m, 2H, phenolic ring), 7.27 (t, 1H, *J* = 7.9 Hz, phenolic ring), 7.99 (d, 2H, *J* = 8.6 Hz, *para*-disubstituted phenyl ring), 8.10 (d, 2H, *J* = 8.7 Hz, *para*-disubstituted phenyl ring), 8.61 (s, 1H, H-triazole). ^13^C-NMR (CD_3_OD) δ (ppm): 29.62 (CH_2_ piperidine), 30.19 (CH_2_ piperidine), 42.81 (CH_2_ piperidine), 44.21 (CH piperidine), 46.13 (CH_2_ piperidine), 51.80 (CH_2_ linker), 62.71 (CH_2_ linker), 69.06 (Glc-CH_2_), 71.56 (Glc), 75.01 (Glc), 78.03 (Glc), 78.11 (Glc), 104.60 (anomeric Glc-C1), 114.46 (CH phenolic ring), 117.94 (CH phenolic ring), 118.52 (CH phenolic ring), 124.59 (CH phenolic ring), 126.85 (2C, *para*-disubstituted phenyl ring), 130.29 (2C, *para*-disubstituted phenyl ring), 130.98 (CH-triazole), 136.44 (C-phenolic ring), 136.69 (*para*-disubstituted phenyl ring), 138.33 (*para*-disubstituted phenyl ring), 147.48 (C-triazole), 158.99 (C-OH phenolic), 172.55 (amidic C=O), 203.22 (ketonic C=O). HPLC analysis (batch FB49 in the Supporting Information): retention time = 11.688; peak area > 99% (254 nm). HRMS: *m*/*z* for C_29_H_33_N_4_O_9_ [M − H]^−^ calculated: 581.22475, found: 581.22504; for C_29_H_34_N_4_O_9_Cl [M + Cl]^−^ calculated: 617.20143, found: 617.20184.

### 3.2. MAGL Inhibition Assays

Compounds **17** and **18**, together with reference compound **13** [46], were evaluated for MAGL inhibition following a protocol previously developed and validated for benzoylpiperidine-based MAGL inhibitors, as reported in the literature [33,44]. Recombinant human MAGL and the substrate 4-nitrophenylacetate (4-NPA) were purchased from Cayman Chemical. IC_50_ determinations were performed in 96-well microtiter plates. The MAGL enzymatic assay was carried out at room temperature in a final volume of 200 μL using 10 mM Tris buffer (pH 7.2) supplemented with 1 mM EDTA and 0.1 mg/mL bovine serum albumin (BSA). For each well, 150 μL of 4-NPA solution (133.3 μM) was added to 10 μL of DMSO containing the appropriate amount of the test compound. The reaction was initiated by adding 40 μL of MAGL (11 ng/well), ensuring a linear enzymatic response over 30 min. Tested compound concentrations ranged from 0.013 to 200 µM. After 30 min, absorbance at 405 nm was recorded using a Victor X3 microplate reader (PerkinElmer, Waltham, MA, USA). Two control reactions were also performed: one lacked the test compounds and one lacked both the compounds and MAGL. IC_50_ values were calculated from experimental data using the Sigmoidal dose–response fitting function of GraphPad Prism software version 9.0. Reported values correspond to the mean of duplicates from three independent experiments. To avoid false positives, a blank measurement was performed for each compound concentration, and the final absorbance was corrected by subtracting the signal generated by all assay components, except MAGL under the same conditions.

### 3.3. FAAH Inhibition Assays

Recombinant human FAAH and AMC arachidonoylamide were purchased from Cayman Chemical. The assay was performed in 96-well microtiter plates at room temperature in 125 mM Tris buffer (pH 9.0) containing 1 mM EDTA and 0.1 mg/mL BSA, in a final volume of 200 μL. Test compounds (dissolved in DMSO) were incubated with the substrate (10 μM), and the reaction was initiated by addition of FAAH (0.9 μg/well). Fluorescence was measured at λ_ex_ = 340 nm and λ_em_ = 460 nm [41]. Blank controls were included, and IC_50_ values were calculated as described for MAGL inhibition assay.

### 3.4. Cell Viability Assays

PANC-1 cells, MiA PaCa-2 and hTERT cells were commercially available and acquired from ATCC (American Type Culture Collection). Cells were seeded in 96-well plates at a density of 2.000 cells per well in 200 µL of high-glucose DMEM (supplemented with 5% FBS, 2 mM glutamine, and 1% Penicillin/streptavidin). All reagents and media for cell culture were acquired from Euroclone. MAGL inhibitor stock solutions were prepared by dissolving them in DMSO (Sigma Aldrich, Darmstadt, Germany) at 250 mM concentration. After 24 h, working solutions of MAGL inhibitors were prepared by diluting the drug in high or low glucose DMEM. The solutions were administered to the cells, keeping the DMSO concentration constant for each test at 0.1%. Serial dilutions were prepared for the low-glucose conditions (100, 50, 25, 5, 1, 0.1, 0.01 µM) and for the high-glucose conditions (0.01, 0.1, 1, 1.9, 3.9, 5, 7.8, 15.6, 25, 31.25, 50, 62.5, 100, 125, 200, 250 µM). Control wells contained the same percentage of DMSO, without the inhibitor. After incubation for 72 h, cell viability was measured using the CellTiter-Blue^®^ Cell Viability Assay (Promega, Madison, Wisconsin, USA) according to the manufacturer’s instructions and reported protocol [58]. Briefly, 20 µL reagent was added to 100 µL culture medium per well and incubated for 2 h at 37 °C. Fluorescence was recorded using 550/15 nm excitation and 590/10 nm emission filters on a BMG Fluostar Omega (BMG) plate reader version 6.30. Experiments were performed in three independent experiments, each in six technical replicates for each condition. Viability was calculated as a percentage of the signal measured for the control, after subtraction of the blank signal arising from well that did not contain cells. GI_50_ values were derived from the collected data by fitting the experimental viability using a nonlinear least-squares Hill model with fixed maximal response (0% growth) and freely varying Hill slope coefficient. GI_50_ values were estimated along with parameter uncertainty (standard deviation) from the covariance matrix of the fit using a Python (v 3.13).

### 3.5. Docking Calculations

The X-ray structure of *h*MAGL (PDB code 5ZUN) [59] energy minimized in explicit water environment, already employed in our previous work [60], was used as receptor for the docking studies of compounds **13** and **17**, which were performed with the AUTODOCK 4.0 software [61]. The ligands were built using the software MolBook Pro [62,63]. The software Autodock Tools was used to automatically identify the torsion angles in the ligand, add the solvent model and determine ligand and protein atomic charges. Gasteiger and Kollmann charges were assigned for ligand and protein, respectively. The docking site used for calculations was defined in such a way as to contain all residues within a 10 Å shell from the reference ligand in the X-ray crystal structure. The energetic maps were calculated using a grid spacing of 0.375 Å and a distance-dependent function of the dielectric constant. The ligand was subjected to 200 runs of AUTODOCK search using the Lamarckian Genetic Algorithm. In particular, for each docking run, 10,000,000 steps of energy evaluations were performed, the number of individuals in the initial population was set to 500 and a maximum of 10,000,000 generations was simulated. An RMS cut-off 2.0 Å was used for pose clustering. All other settings were left as their default. The best docked conformation belonging to the best cluster of solutions obtained (top-scored pose) was considered for each ligand.

## 4. Conclusions

This study explored a glucose-conjugation strategy to potentially improve the cellular delivery profile of *N*-benzoylpiperidine MAGL inhibitors in metabolically rewired cancer cells. Using a modular synthetic sequence that culminates in a CuAAC coupling between an alkyne-bearing benzoylpiperidine core and azido-functionalized glucopyranose units, two triazole-linked glycoconjugates were obtained (compounds **17** and **18**). The relatively low yields observed in the final CuAAC step likely reflect the lack of specific optimization of the click reaction conditions, which were deliberately kept general and exploratory at this proof-of-concept stage. Reaction yields could be improved in the future by adjusting reaction conditions, exploring different catalysts, temporarily protecting the sugar hydroxyl groups or by investigating microwave-assisted reaction conditions.

Despite the increased polarity introduced by the sugar moiety, both derivatives retained measurable inhibitory activity against human MAGL, with mid-micromolar IC_50_ values (43.3–68.8 μM). Within this limited set, changing the glucopyranose conjugation pattern did not markedly impact enzyme inhibition, indicating that glycosylation is broadly compatible with the scaffold, but it comes with a clear potency penalty relative to the parent reference inhibitor.

In contrast, the in vitro data obtained in the cell-based experiments revealed a pronounced dependence on extracellular glucose. While the glycoconjugates were inactive in PANC-1 cells under high-glucose conditions (GI_50_ > 1000 μM), they displayed antiproliferative activity in low-glucose medium, with one compound (**18**) reaching low-micromolar potency (GI_50_ = 12 μM). Future studies will be performed to further investigate this discrepancy and to exclude possible off-target effects or non-MAGL-mediated mechanisms. This behavior is consistent with the working hypothesis that the carbohydrate appendage can modulate cellular uptake and that high glucose may competitively limit internalization. Overall, these results introduce, to the best of our knowledge, the first glycol-conjugated inhibitors specifically designed to target MAGL. Although the glycol-conjugation strategy resulted in a reduction in enzymatic potency compared to the reference inhibitor, the mid-micromolar inhibition observed remains biologically meaningful in the context of a proof-of-concept study aimed at modulating cellular activity through metabolism-dependent mechanisms. It is worth noting that the biological findings reported herein should be regarded as preliminary with respect to the mechanistic interpretation. Further in-depth studies will be necessary to clarify the precise cellular uptake pathways and to confirm the contribution of MAGL inhibition versus potential off-target effects in the observed antiproliferative activity. Computational analyses suggest that steric and conformational constraints introduced by the sugar moiety likely affect optimal positioning within the catalytic site, thereby providing a rational basis for the observed decrease in activity. Importantly, these insights offer clear directions for further optimization, including fine-tuning of the linker length and flexibility, modulation of sugar substitution patterns, and exploration of alternative glycosidic configurations to improve binding interactions while preserving favorable cellular profile and selectivity. Collectively, these findings establish a foundation for the rational development of next-generation glycol-conjugated MAGL inhibitors with enhanced enzymatic potency and retained metabolism-driven activity.

## Figures and Tables

**Table 1:** In vitro inhibitory activity on human MAGL (*h*MAGL, IC_50_, µM) ^a^ of glycoconjugated MAGL inhibitors **17**, **18** and reference compound **13**.

**Compound**	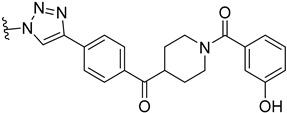	**IC_50_ *h*MAGL** **(Mean ± SD, µM)**
**17**	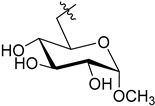	43.3 ± 0.6
**18**	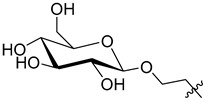	68.8 ± 3.6
**13** ^b^	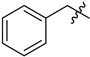	4.1 ± 0.7

^a^ Enzymatic values are the mean of three independent experiments, performed in duplicate, ±SD. ^b^ Ref. [46].

**Table 2:** In vitro antiproliferative activity ^a^ of glycoconjugated MAGL inhibitors **17**, **18** and reference compounds **13**.

Compound	GI_50_ PANC-1 High Glucose	GI_50_ PANC-1 Low Glucose	GI_50_ MiA PaCa-2	GI_50_ Fibroblast Low Glucose
	(Mean ± SD, µM)	(Mean ± SD, µM)	(Mean ± SD, µM)	(mean ± SD, µM)
**17**	>1000	129 ± 46	>1000	>1000
**18**	>1000	12 ± 9	>1000	>1000
**13** ^b^	15 ± 14	4 ± 3	420 ± 90	33 ± 10

^a^ Viability values are the mean of three independent experiments, performed in six technical replicates for each concentration, using seven different concentrations for analyses in low-glucose conditions and 16 different concentrations for analyses in high-glucose conditions (see Section 3 Materials and Methods for further information regarding the concentration used). Data are expressed as mean ± SD. ^b^ Ref [46].

## Data Availability

The original contributions presented in this study are included in the article/Supplementary Material. Further inquiries can be directed to the corresponding author.

## Supplementary Materials

The following supporting information can be downloaded at https://www.mdpi.com/article/10.3390/ijms27062666/s1.

## Abbreviations

The following abbreviations are used in this manuscript:

2-AG	2-Arachidonoylglycerol
CuAAC	Copper-catalyzed azide–alkyne cycloaddition
DIPEA	*N*,*N*-Diisopropylethylamine
DMF	*N*,*N*-Dimethylformamide
GLUT	Glucose transporter
HATU	1-[Bis(dimethylamino)methylene]-1*H*-1,2,3-triazolo[4,5-*b*]pyridinium-3-oxide hexafluorophosphate
MAGL	Monoacylglycerol lipase
MEBYNOL	2-Methyl-3-butyn-2-ol
MEM	Methoxyethoxymethyl
4-NPA	4-Nitrophenylacetate
SD	Standard deviation

## References

[1] Seyfried T.N., Flores R.E., Poff A.M., D’Agostino D.P. (2014). Cancer as a Metabolic Disease: Implications for Novel Therapeutics. Carcinogenesis.

[2] Cairns R.A., Harris I.S., Mak T.W. (2011). Regulation of Cancer Cell Metabolism. Nat. Rev. Cancer.

[3] Pavlova N.N., Thompson C.B. (2016). The Emerging Hallmarks of Cancer Metabolism. Cell Metab..

[4] Vazquez A., Kamphorst J.J., Markert E.K., Schug Z.T., Tardito S., Gottlieb E. (2016). Cancer Metabolism at a Glance. J. Cell Sci..

[5] Vander Heiden M.G., Cantley L.C., Thompson C.B. (2009). Understanding the Warburg Effect: The Metabolic Requirements of Cell Proliferation. Science.

[6] Warburg O. (1956). On Respiratory Impairment in Cancer Cells. Science.

[7] Snaebjornsson M.T., Janaki-Raman S., Schulze A. (2020). Greasing the Wheels of the Cancer Machine: The Role of Lipid Metabolism in Cancer. Cell Metab..

[8] Chen Y., Li P. (2016). Fatty Acid Metabolism and Cancer Development. Sci. Bull..

[9] Cheng C., Geng F., Cheng X., Guo D. (2018). Lipid Metabolism Reprogramming and Its Potential Targets in Cancer. Cancer Commun..

[10] Warburg O. (1956). On the Origin of Cancer Cells. Science.

[11] Warburg O., Wind F., Negelein E. (1927). The Metabolism of Tumors in the Body. J. Gen. Physiol..

[12] Fortunato S., Bononi G., Granchi C., Minutolo F. (2018). An Update on Patents Covering Agents That Interfere with the Cancer Glycolytic Cascade. ChemMedChem.

[13] Yuan Q., Liu H., Song C., Tian Y., Hassan W., Shabbir R., Wenjuan C., Han L. (2025). Prevalence of GLUT1 Overexpression in Human Cancers a Systematic Review and Meta Analysis. Discov. Oncol..

[14] Franczak M., Kutryb-Zajac B., El Hassouni B., Giovannetti E., Granchi C., Minutolo F., Smolenski R.T., Peters G.J. (2022). The Effect of Lactate Dehydrogenase-A Inhibition on Intracellular Nucleotides and Mitochondrial Respiration in Pancreatic Cancer Cells. Nucleosides Nucleotides Nucleic Acids.

[15] Takahashi M., Nojima H., Kuboki S., Horikoshi T., Yokota T., Yoshitomi H., Furukawa K., Takayashiki T., Takano S., Ohtsuka M. (2020). Comparing Prognostic Factors of Glut-1 Expression and Maximum Standardized Uptake Value by FDG-PET in Patients with Resectable Pancreatic Cancer. Pancreatology.

[16] Kawatani M., Aono H., Hiranuma S., Shimizu T., Muroi M., Ogawa N., Ohishi T., Ohba S., Kawada M., Nogawa T. (2021). Identification of a Small-Molecule Glucose Transporter Inhibitor, Glutipyran, That Inhibits Cancer Cell Growth. ACS Chem. Biol..

[17] Bononi G., Iacopini D., Cicio G., Di Pietro S., Granchi C., Di Bussolo V., Minutolo F. (2021). Glycoconjugated Metal Complexes as Cancer Diagnostic and Therapeutic Agents. ChemMedChem.

[18] Brescia F., Titilas I., Cacciapuoti S., Ronconi L. (2025). Recent Advances in the Development of Metal-Glycoconjugates for Medicinal Applications. Molecules.

[19] Fu J., Yang J., Seeberger P.H., Yin J. (2020). Glycoconjugates for Glucose Transporter-Mediated Cancer-Specific Targeting and Treatment. Carbohydr. Res..

[20] Chen L.-L., Wang W.-J. (2021). P53 Regulates Lipid Metabolism in Cancer. Int. J. Biol. Macromol..

[21] Mukherjee S., Maxfield F.R. (2004). Membrane Domains. Annu. Rev. Cell Dev. Biol..

[22] Pomorski T., Hrafnsdóttir S., Devaux P.F., van Meer G. (2001). Lipid Distribution and Transport across Cellular Membranes. Semin. Cell Dev. Biol..

[23] van Meer G. (2010). Membranes in Motion. EMBO Rep..

[24] van Meer G., Voelker D.R., Feigenson G.W. (2008). Membrane Lipids: Where They Are and How They Behave. Nat. Rev. Mol. Cell Biol..

[25] Holthuis J.C.M., Menon A.K. (2014). Lipid Landscapes and Pipelines in Membrane Homeostasis. Nature.

[26] Zechner R., Zimmermann R., Eichmann T.O., Kohlwein S.D., Haemmerle G., Lass A., Madeo F. (2012). FAT SIGNALS—Lipases and Lipolysis in Lipid Metabolism and Signaling. Cell Metab..

[27] Nomura D.K., Long J.Z., Niessen S., Hoover H.S., Ng S.-W., Cravatt B.F. (2010). Monoacylglycerol Lipase Regulates a Fatty Acid Network That Promotes Cancer Pathogenesis. Cell.

[28] Mulvihill M.M., Nomura D.K. (2013). Therapeutic Potential of Monoacylglycerol Lipase Inhibitors. Life Sci..

[29] Gil-Ordóñez A., Martín-Fontecha M., Ortega-Gutiérrez S., López-Rodríguez M.L. (2018). Monoacylglycerol Lipase (MAGL) as a Promising Therapeutic Target. Biochem. Pharmacol..

[30] Deng H., Li W. (2020). Monoacylglycerol Lipase Inhibitors: Modulators for Lipid Metabolism in Cancer Malignancy, Neurological and Metabolic Disorders. Acta Pharm. Sin. B.

[31] King A.R., Duranti A., Tontini A., Rivara S., Rosengarth A., Clapper J.R., Astarita G., Geaga J.A., Luecke H., Mor M. (2007). URB602 Inhibits Monoacylglycerol Lipase and Selectively Blocks 2-Arachidonoylglycerol Degradation in Intact Brain Slices. Chem. Biol..

[32] Long J.Z., Li W., Booker L., Burston J.J., Kinsey S.G., Schlosburg J.E., Pavón F.J., Serrano A.M., Selley D.E., Parsons L.H. (2009). Selective Blockade of 2-Arachidonoylglycerol Hydrolysis Produces Cannabinoid Behavioral Effects. Nat. Chem. Biol..

[33] Muccioli G.G., Labar G., Lambert D.M. (2008). CAY10499, a Novel Monoglyceride Lipase Inhibitor Evidenced by an Expeditious MGL Assay. ChemBioChem.

[34] Cisar J.S., Weber O.D., Clapper J.R., Blankman J.L., Henry C.L., Simon G.M., Alexander J.P., Jones T.K., Ezekowitz R.A.B., O’Neill G.P. (2018). Identification of ABX-1431, a Selective Inhibitor of Monoacylglycerol Lipase and Clinical Candidate for Treatment of Neurological Disorders. J. Med. Chem..

[35] Schlosburg J.E., Blankman J.L., Long J.Z., Nomura D.K., Pan B., Kinsey S.G., Nguyen P.T., Ramesh D., Booker L., Burston J.J. (2010). Chronic Monoacylglycerol Lipase Blockade Causes Functional Antagonism of the Endocannabinoid System. Nat. Neurosci..

[36] Schlosburg J.E., Kinsey S.G., Ignatowska-Jankowska B., Ramesh D., Abdullah R.A., Tao Q., Booker L., Long J.Z., Selley D.E., Cravatt B.F. (2014). Prolonged Monoacylglycerol Lipase Blockade Causes Equivalent Cannabinoid Receptor Type 1 Receptor–Mediated Adaptations in Fatty Acid Amide Hydrolase Wild-Type and Knockout Mice. J. Pharmacol. Exp. Ther..

[37] King A.R., Dotsey E.Y., Lodola A., Jung K.M., Ghomian A., Qiu Y., Fu J., Mor M., Piomelli D. (2009). Discovery of Potent and Reversible Monoacylglycerol Lipase Inhibitors. Chem. Biol..

[38] Chicca A., Marazzi J., Gertsch J. (2012). The Antinociceptive Triterpene Β-amyrin Inhibits 2-arachidonoylglycerol (2-AG) Hydrolysis without Directly Targeting Cannabinoid Receptors. Br. J. Pharmacol..

[39] Chevalier K.M., Dax S.L., Flores C.M., Liu L., Macielag M.J., McDonnel M.E., Nelen M.I., Prouty S., Todd M., Zhang S. (2010). Heteroaromatic and Aromatic Piperazinyl Azetidinyl Amides as Monoacylglycerol Lipase Inhibitors.

[40] Granchi C., Lapillo M., Glasmacher S., Bononi G., Licari C., Poli G., el Boustani M., Caligiuri I., Rizzolio F., Gertsch J. (2019). Optimization of a Benzoylpiperidine Class Identifies a Highly Potent and Selective Reversible Monoacylglycerol Lipase (MAGL) Inhibitor. J. Med. Chem..

[41] Bononi G., Tonarini G., Poli G., Barravecchia I., Caligiuri I., Macchia M., Rizzolio F., Demontis G.C., Minutolo F., Granchi C. (2021). Monoacylglycerol Lipase (MAGL) Inhibitors Based on a Diphenylsulfide-Benzoylpiperidine Scaffold. Eur. J. Med. Chem..

[42] Di Stefano M., Masoni S., Bononi G., Poli G., Galati S., Gado F., Manzi S., Vagaggini C., Brai A., Caligiuri I. (2024). Design, Synthesis, ADME and Biological Evaluation of Benzylpiperidine and Benzylpiperazine Derivatives as Novel Reversible Monoacylglycerol Lipase (MAGL) Inhibitors. Eur. J. Med. Chem..

[43] Jiang M., Huizenga M.C.W., Wirt J.L., Paloczi J., Amedi A., van den Berg R.J.B.H.N., Benz J., Collin L., Deng H., Di X. (2023). A Monoacylglycerol Lipase Inhibitor Showing Therapeutic Efficacy in Mice without Central Side Effects or Dependence. Nat. Commun..

[44] Tuccinardi T., Granchi C., Rizzolio F., Caligiuri I., Battistello V., Toffoli G., Minutolo F., Macchia M., Martinelli A. (2014). Identification and Characterization of a New Reversible MAGL Inhibitor. Bioorg. Med. Chem..

[45] Granchi C., Rizzolio F., Palazzolo S., Carmignani S., Macchia M., Saccomanni G., Manera C., Martinelli A., Minutolo F., Tuccinardi T. (2016). Structural Optimization of 4-Chlorobenzoylpiperidine Derivatives for the Development of Potent, Reversible, and Selective Monoacylglycerol Lipase (MAGL) Inhibitors. J. Med. Chem..

[46] Granchi C., Bononi G., Ferrisi R., Gori E., Mantini G., Glasmacher S., Poli G., Palazzolo S., Caligiuri I., Rizzolio F. (2021). Design, Synthesis and Biological Evaluation of Second-Generation Benzoylpiperidine Derivatives as Reversible Monoacylglycerol Lipase (MAGL) Inhibitors. Eur. J. Med. Chem..

[47] Bononi G., Lonzi C., Tuccinardi T., Minutolo F., Granchi C. (2024). The Benzoylpiperidine Fragment as a Privileged Structure in Medicinal Chemistry: A Comprehensive Review. Molecules.

[48] Calvaresi E.C., Hergenrother P.J. (2013). Glucose Conjugation for the Specific Targeting and Treatment of Cancer. Chem. Sci..

[49] Lee A.A., Chen Y.S., Ekalestari E., Ho S., Hsu N., Kuo T., Wang T.A. (2016). Facile and Versatile Chemoenzymatic Synthesis of Enterobactin Analogues and Applications in Bacterial Detection. Angew. Chem. Int. Ed..

[50] Bose P., Singh M., Gupta A., Kumar S., Ansari F.J., Pandey V.K., Singh A.S., Tiwari V.K. (2024). Design, Synthesis, and Docking Study of Saccharin N-Triazolyl Glycoconjugates. Carbohydr. Res..

[51] Lamandé-Langle S., Collet C., Hensienne R., Vala C., Chrétien F., Chapleur Y., Mohamadi A., Lacolley P., Regnault V. (2014). ‘Click’ Glycosylation of Peptides through Cysteine Propargylation and CuAAC. Bioorg. Med. Chem..

[52] Iacopini D., Vančo J., Di Pietro S., Bordoni V., Zacchini S., Marchetti F., Dvořák Z., Malina T., Biancalana L., Trávníček Z. (2022). New Glycoconjugation Strategies for Ruthenium(II) Arene Complexes via Phosphane Ligands and Assessment of Their Antiproliferative Activity. Bioorg. Chem..

[53] Daemen A., Peterson D., Sahu N., McCord R., Du X., Liu B., Kowanetz K., Hong R., Moffat J., Gao M. (2015). Metabolite Profiling Stratifies Pancreatic Ductal Adenocarcinomas into Subtypes with Distinct Sensitivities to Metabolic Inhibitors. Proc. Natl. Acad. Sci. USA.

[54] Montesdeoca N., López M., Ariza X., Herrero L., Makowski K. (2020). Inhibitors of Lipogenic Enzymes as a Potential Therapy against Cancer. FASEB J..

[55] Tabatabaei P., Visse E., Bergström P., Brännström T., Siesjö P., Bergenheim A.T. (2017). Radiotherapy Induces an Immediate Inflammatory Reaction in Malignant Glioma: A Clinical Microdialysis Study. J. Neurooncol..

[56] Rossi G., Petrone M.C., Tacelli M., Zaccari P., Crippa S., Belfiori G., Aleotti F., Locatelli M., Piemonti L., Doglioni C. (2024). Glucose and Lactate Levels Are Lower in EUS-Aspirated Cyst Fluid of Mucinous vs Non-Mucinous Pancreatic Cystic Lesions. Dig. Liver Dis..

[57] Noia J.L., Mejuto R., Oria I., De la Iglesia-García D., Villaverde A., Voces A., Pizzala J., Iglesias-García J., Urgiles D., Marcolongo M. (2022). Rapid Diagnosis of Mucinous Cystic Pancreatic Lesions by On-Site Cyst Fluid Glucometry. Surg. Endosc..

[58] O’Brien J., Wilson I., Orton T., Pognan F. (2000). Investigation of the Alamar Blue (Resazurin) Fluorescent Dye for the Assessment of Mammalian Cell Cytotoxicity. Eur. J. Biochem..

[59] Aida J., Fushimi M., Kusumoto T., Sugiyama H., Arimura N., Ikeda S., Sasaki M., Sogabe S., Aoyama K., Koike T. (2018). Design, Synthesis, and Evaluation of Piperazinyl Pyrrolidin-2-Ones as a Novel Series of Reversible Monoacylglycerol Lipase Inhibitors. J. Med. Chem..

[60] Bononi G., Di Stefano M., Poli G., Ortore G., Meier P., Masetto F., Caligiuri I., Rizzolio F., Macchia M., Chicca A. (2022). Reversible Monoacylglycerol Lipase Inhibitors: Discovery of a New Class of Benzylpiperidine Derivatives. J. Med. Chem..

[61] Morris G.M., Huey R., Lindstrom W., Sanner M.F., Belew R.K., Goodsell D.S., Olson A.J. (2009). AutoDock4 and AutoDockTools4: Automated Docking with Selective Receptor Flexibility. J. Comput. Chem..

[62] Galati S., Di Stefano M., Macchia M., Poli G., Tuccinardi T. (2023). MolBook UNIPI─Create, Manage, Analyze, and Share Your Chemical Data for Free. J. Chem. Inf. Model..

[63] Galati S., Di Stefano M., Piazza L., Poles C., Macchia M., Tuccinardi T. (2025). MolBook Pro. https://molbookpro.farm.unipi.it/.

