# Supplementary material for: Development of Glycoconjugated MAGL Inhibitors with Glucose-Dependent Antiproliferative Activity
Source: Int J Mol Sci. 2026 Mar 14;27(6):2666. doi: 10.3390/ijms27062666 (PMC13027055; doi:10.3390/ijms27062666)
Supplement: Supplementary file 1 [file ijms-27-02666-s001.zip › ijms-4129006-supplementary.pdf]

Supporting Information

Development of Glycoconjugated MAGL Inhibitors with Glucose-Dependent Antiproliferative Activity

Giulia Bononi[a,b], Federica Bertini[a], Samuele Masoni[a], Miriana Di Stefano[a], Rossella Mosca[c], Francesca Felice[c], Giovanni Signore[c,d], Filippo Minutolo[a,b]. Carlotta Granchi[a,b,], Tiziano Tuccinardi[a,b], Valeria Di Bussolo[a]*

[a] Department of Pharmacy, University of Pisa, Via Bonanno 6, 56126 Pisa, Italy.

[b] Center for Instrument Sharing of the University of Pisa (CISUP), Lungarno Pacinotti 43, 56126 Pisa, Italy.

[c] Department of Biology, Biochemistry Unit, University of Pisa, Via S. Zeno 51, 56127 Pisa, Italy.

[d] Institute of Clinical Physiology, National Research Council, 56124 Pisa, Italy.

Table of Contents

Figure S1-S2. RP-HPLC traces of final compounds. S2-S3

Figure S3-S4. ^{1}H- and ^{13}C-NMR spectra of final compounds. S4-S5

Figure S5-S6. ESI-HRMS spectra of final compounds. S6

==== Shimadzu LabSolutions Analysis Report ====

Sample Name : FB36_500uM_4
Sample ID : FB36_500uM_4
Data Filename : FB36_500uM_4_p_c.lcd
Method Filename : MAGL254_prova_2.lcm

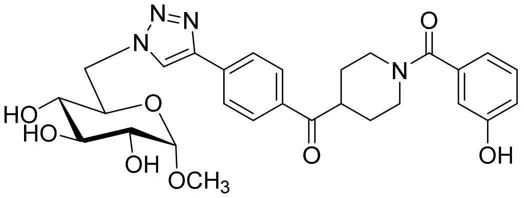

17

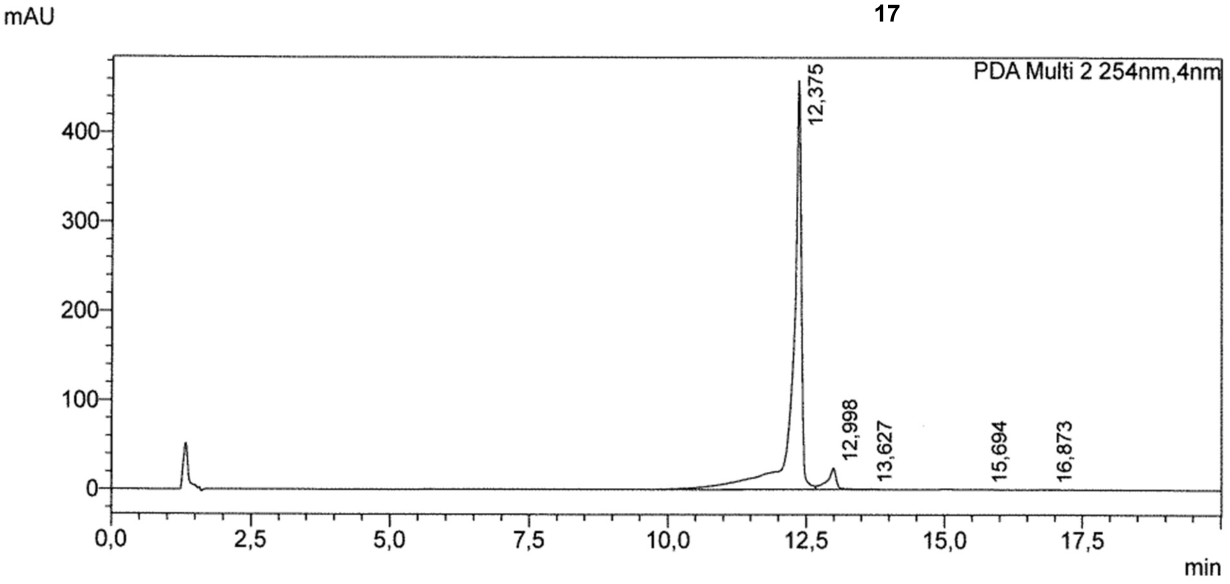

PDA Ch2 254nm

Peak#	Ret. Time	Area	Area%	Height
1	12,375	4805477	94,529	457981
2	12,998	271808	5,347	23764
3	13,627	1253	0,025	189
4	15,694	1942	0,038	185
5	16,873	3127	0,062	193
Total		5083607	100,000	482313

Figure S1. HPLC chromatogram of compound **17**.

==== Shimadzu LabSolutions Analysis Report ====

Sample Name : FB49_500uM
Sample ID : FB49_500uM
Data Filename : FB49_500uM_p_c.lcd
Method Filename : MAGL254 prova 2.lcm

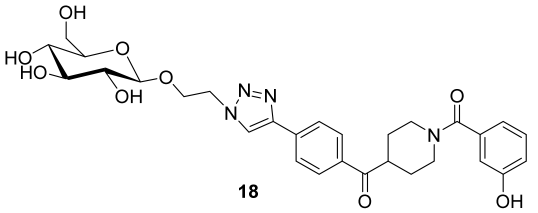

18

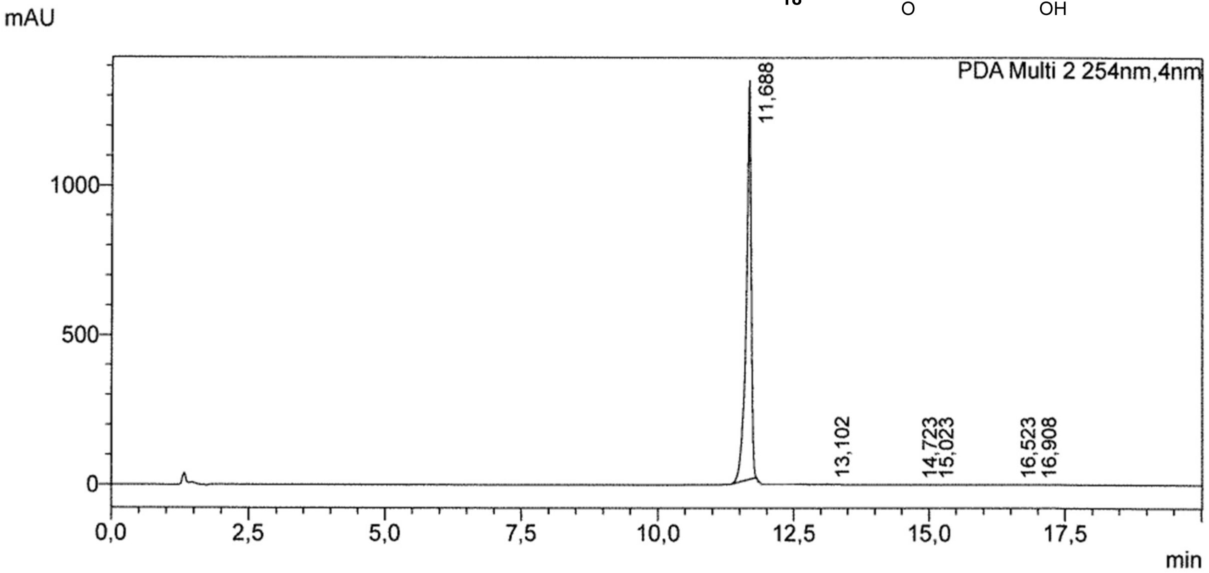

PDA Ch2 254nm

Peak#	Ret. Time	Area	Area%	Height
1	11,688	8501217	99,650	1333859
2	13,102	7121	0,083	1043
3	14,723	8319	0,098	396
4	15,023	9461	0,111	436
5	16,523	1291	0,015	80
6	16,908	3638	0,043	247
Total		8531047	100,000	1336061

Figure S2. HPLC chromatogram of compound **18**.

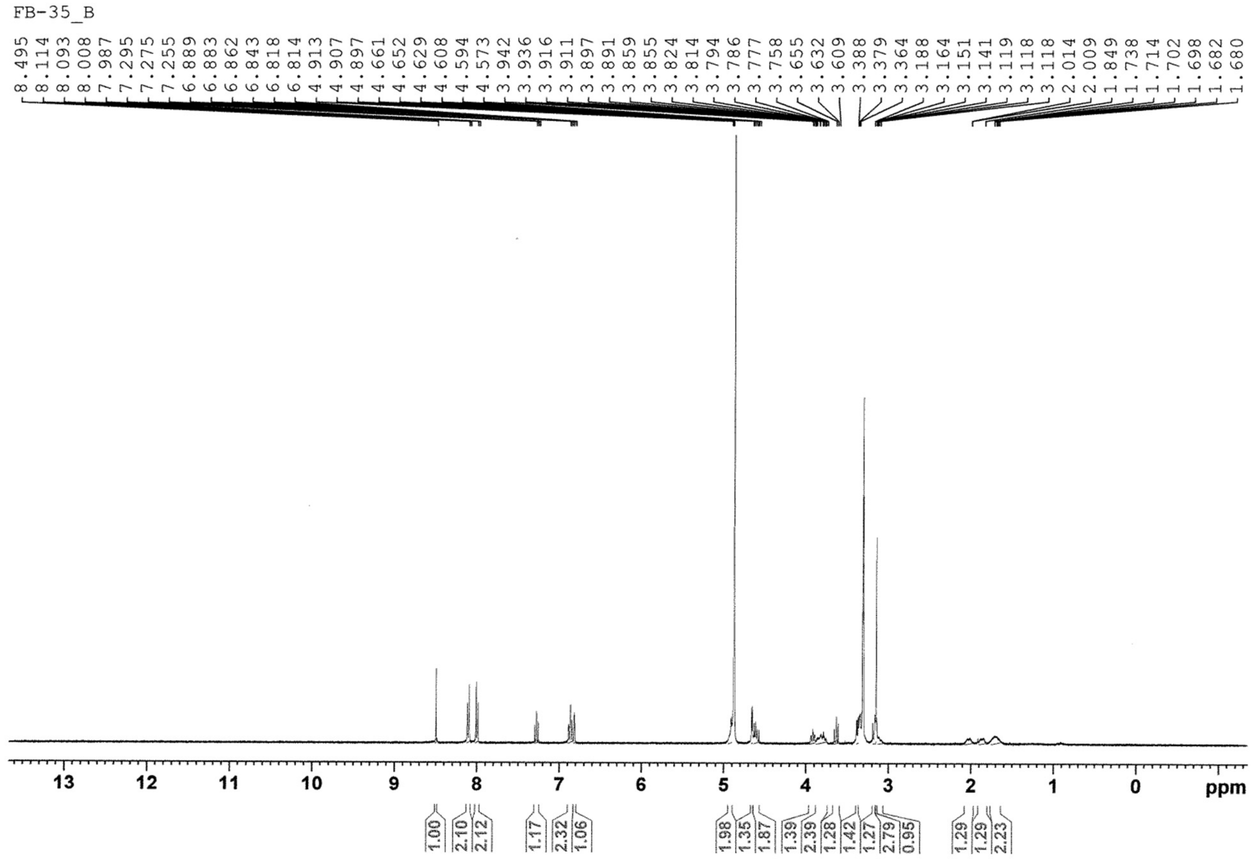

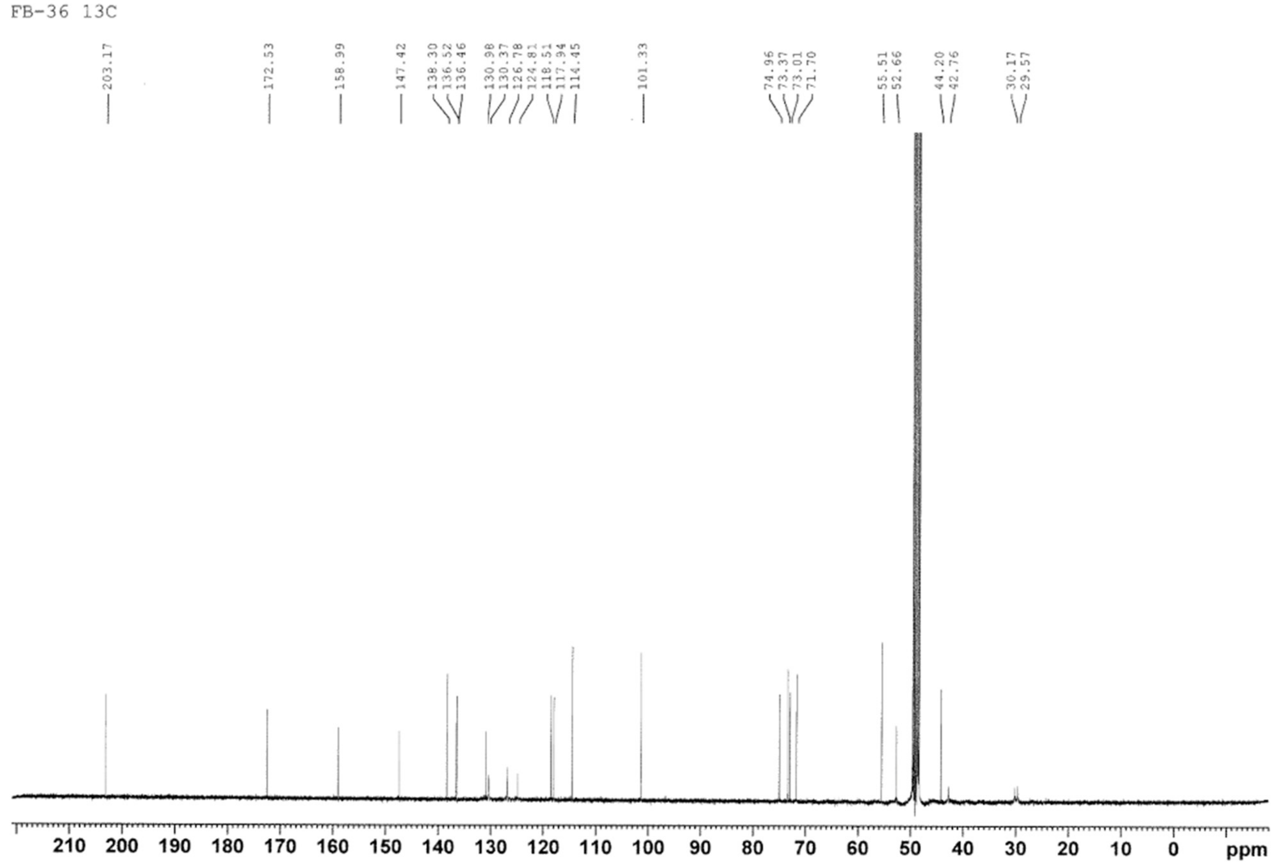

Figure S3. ^{1}H-NMR and ^{13}C-NMR of compound **17**.

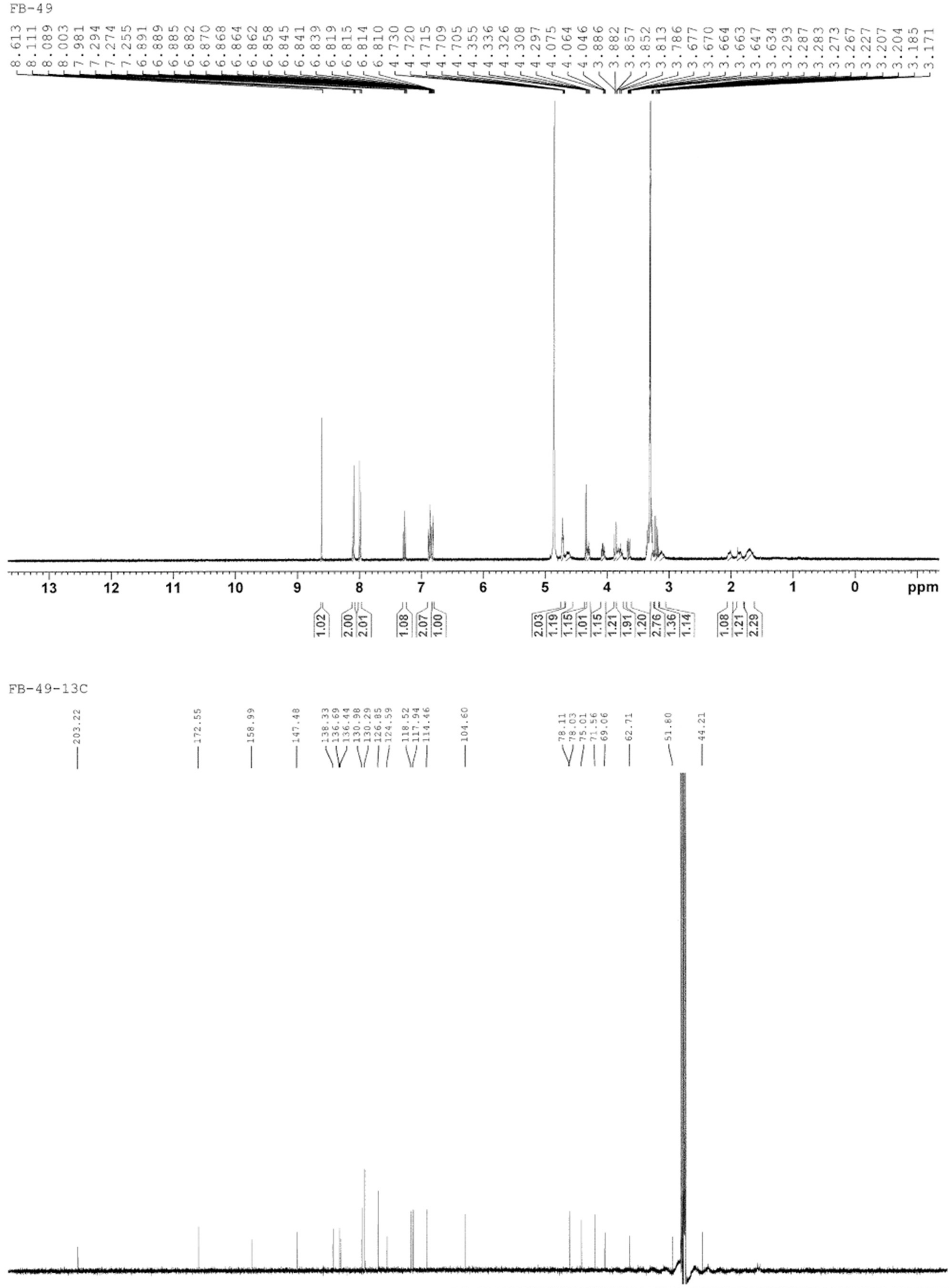

Figure S4. ^{1}H-NMR and ^{13}C-NMR of compound **18**.

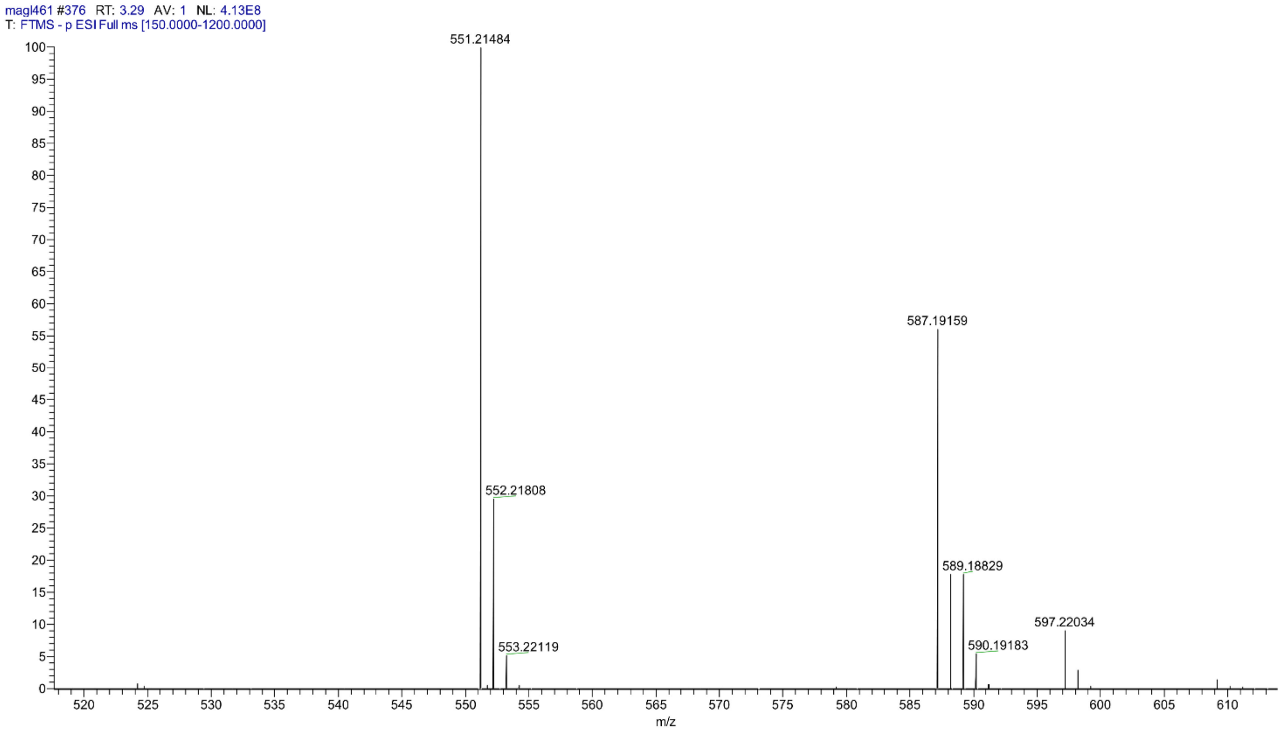

Figure S5. ESI-HRMS spectrum of compound **17**.

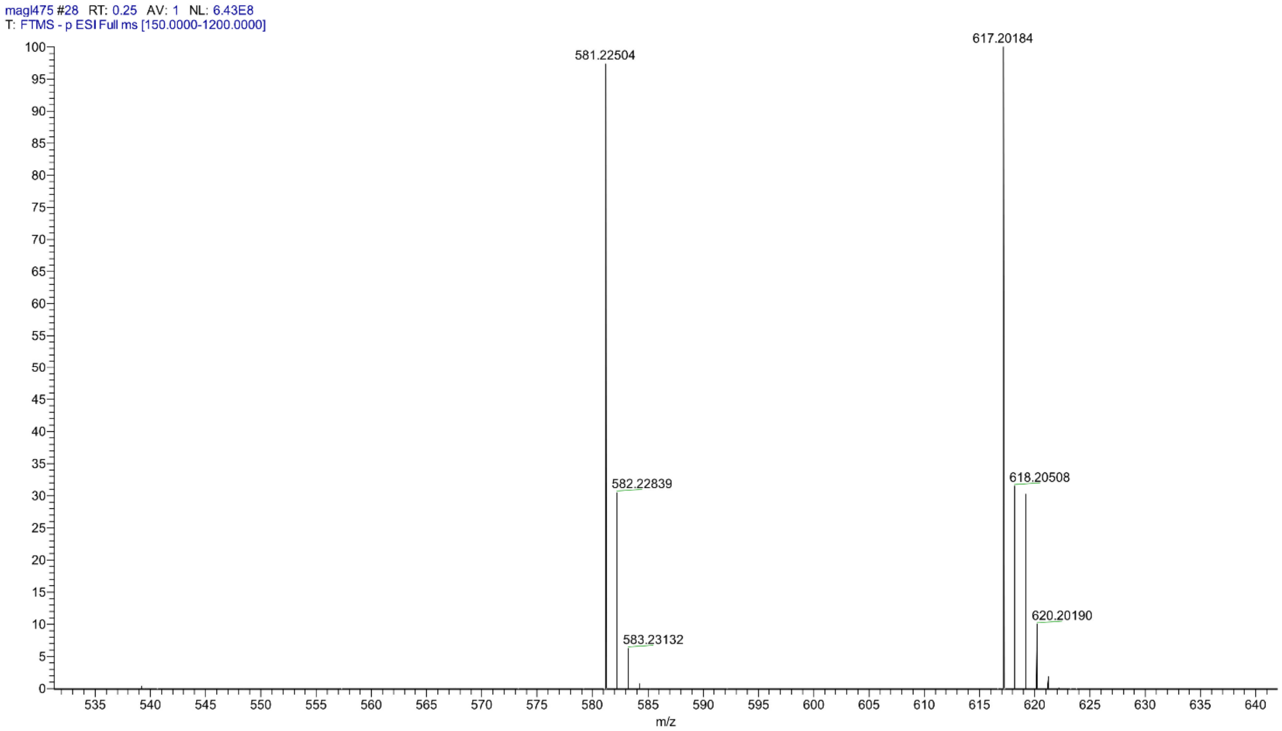

Figure S6. ESI-HRMS spectrum of compound **18**.
